# p53–GSDME Elevation: A Path for CDK7 Inhibition to Suppress Breast Cancer Cell Survival

**DOI:** 10.3389/fmolb.2021.697457

**Published:** 2021-08-19

**Authors:** Yueyuan Wang, Jingyu Peng, Xuguang Mi, Ming Yang

**Affiliations:** ^1^ Department of Breast Surgery, The First Hospital of Jilin University, Changchun, China; ^2^ Tumor Biotherapy Center, Jilin Province People’s Hospital, Changchun, China

**Keywords:** CDK7, p53, GSDME, CDK7 inhibitor, breast cancer

## Abstract

Higher cyclin-dependent kinase (CDK7) expression is a character of breast cancer and indicates poor prognosis. Inhibiting CDK7 exhibited effective cancer cell suppression which implies the potential of CDK7 inhibition to be a method for anti-cancer treatment. Our study aimed to explore a novel mechanism of CDK7 inhibition for suppressing breast cancer cell survival. Here, we proved inhibiting CDK7 repressed breast cancer cell proliferation and colony formation and increased the apoptotic cell rate, with p53 and GSDME protein level elevation. When p53 was suppressed in MCF-7 cells, the decline of GSDME expression and associated stronger proliferation and colony formation could be observed. Since downregulation of GSDME was of benefit to breast cancer cells, p53 inhibition blocked the elevation of GSDME induced by CDK7 inhibition and retrieved cells from the tumor suppressive effect of CDK7 inhibition. Therefore, CDK7 inhibition exerted a negative effect on breast cancer cell proliferation and colony formation in a p53–GSDME dependent manner. These results revealed the CDK7–p53–GSDME axis could be a pathway affecting breast cancer cell survival.

## Introduction

Female breast cancer is the most diagnosed cancer (11.7%), with an estimated 2.3 million new cases according to Global Cancer Statics 2020 (Sung et al., 2021). Compared with traditional therapeutic methods such as surgical excision, chemotherapy, and radiotherapy, molecular targeted therapy provides better specificity and fewer side effects. Although the molecular mechanisms of breast tumorigenesis are still unclear, the upregulation of genes that modulate the mitotic cell cycle was identified by bioinformation analysis (Deng et al., 2019). In particular, overexpression of cyclin-dependent kinase 7 (CDK7) has been reported in breast cancer and is highly associated with poor prognosis (Patel et al., 2016; Li et al., 2017). Moreover, CDK7 expression is positively correlated with that of the estrogen receptor (ER) and plays a role in phosphorylating Ser118 on the ER, which promotes ER activity, and CDK7 blockade contributes to reverse endocrine therapy resistance in breast cancer (Patel et al., 2016; Attia et al., 2020). Disruption of transcriptional addiction to a vital cluster of genes in triple negative breast cancer (TNBC) by CDK7 inhibition was revealed by Gray and Zhao’s group (Wang et al., 2015). Because CDK7 transcriptional kinase activity is regulated by HER2 and a series of receptor tyrosine kinases activated by HER2 inhibition, and by the downstream SHP2 and PI3K/AKT pathway, dual HER2 and CDK7 inhibition can overcome the therapy resistance of HER2-positive breast cancer and induce significant tumor regression *in vivo* (Sun et al., 2020). Cyclin-dependent kinase 4/6 (CDK4/6) inhibitors for breast cancer treatment have been approved for use in clinical cancer therapeutics. CDK7 inhibitors exert a satisfactory anti-tumor effect on multiple varieties of tumors *in vitro* and *in vivo*, which strongly implies that CDK7 inhibition provides a potential clinical application.

Cell mitosis is a highly regulated process that depends on cell cycle checkpoints, and dysregulation of the cell cycle is a classic hallmark of cancer growth and metastatic potential (Mayer, 2015; VanArsdale et al., 2015). CDKs are catalytic subunits that respond to a large family of serine/threonine protein kinases (Ali et al., 2009). In mammals, CDK7 binds cyclin H and MAT1 to form CDK-activating kinase (CAK), mediating the activation of other CDKs, including CDK1, CDK2, CDK4, and CDK6 (Fisher, 2012). CDK7 is also associated with the general transcription factor IIH (TFIIH) and phosphorylates RNA polymerase (RNAP) II, p53, and other transcription factors, including retinoid receptors, the androgen receptor, and the ER (Akhtar et al., 2009; Larochelle et al., 2012; Schachter and Fisher, 2013; Patel et al., 2016). High ectopic expression of CDK7 has been detected in multiple types of cancers, including gastric cancer, oral squamous cell carcinoma, and breast cancer, and is associated with aggressive clinicopathological features and poor prognosis (Wang et al., 2016a; Li et al., 2017; Jiang et al., 2019; Diab et al., 2020).

THZ1 and LDC4297 are two small inhibitors with high CDK7 affinity but function along different pathways. THZ1 inhibits CDK7 by targeting cysteine located outside the kinase domain and occupying it covalently. As a low nanomolar inhibitor of cell proliferation and CDK7 activity, THZ1 inhibits CDK12 and CDK13 kinase activity at higher concentrations (Kwiatkowski et al., 2014). THZ1 affects multiple cancer cells by impairing the transcription of super-enhancers and several small subsets of genes that induce cell cycle arrest and apoptosis (Christensen et al., 2014; Kwiatkowski et al., 2014; Wang et al., 2015; Zhong et al., 2018; Lu et al., 2019). LDC4297 inhibits CDK7 efficiently *in vitro* in the nano-picomolar range (Hutterer et al., 2015; Wang et al., 2015).

Gasdermin E (GSDME), of which mutant form was long considered to relate to hereditary hearing impairment, was named human deafness autosomal dominant 5 (DFNA5) on gene level, and GSDME expression is significantly lower in ER-positive breast carcinomas than in ER-negative breast carcinomas (Thompson and Weigel, 1998). Furthermore, GSDME expression is repressed by promoter hypermethylation in gastric, breast, and colorectal cancers, with 52–65% of primary tumors achieving epigenetic silencing. High-frequency GSDME methylation in breast cancer patients indicates a worse five-year survival rate and a higher lymph node metastasis incidence (Akino et al., 2007; Kim et al., 2008a; Kim et al., 2008b; Croes et al., 2018). Moreover, caspase-3 cleavage of *GSDME* in chemotherapy mediates anti-tumor immunity (Rogers et al., 2017; Wang et al., 2017; Zhang et al., 2020), substantiating GSDME’s role as a tumor suppressor gene.

The tumor suppressor and nuclear phosphoprotein p53 modulates numerous downstream target genes that regulate cell cycle progression and cell death mediation. p53 surveys DNA integrity and binds damaged DNA, triggering downstream gene p21 transcription, producing G1 arrest, and repressing desmolase activity during DNA repair. If repair fails, p53 initiates apoptosis. p53 is identified as a nuclear phosphoprotein, of which phosphorylation can be a choice for its activation. CDK7–cyclin H is reported to provide efficient phosphorylation of p53 (Ko et al., 1997), and intron 1 of DFNA5 contains a potential p53-binding sequence (Masuda et al., 2006).

Here, we investigated whether a modulation relationship exists among CDK7, p53, and GSDME and whether this relationship is a possible target for inhibiting CDK7 and affecting breast cancer cell survival. We demonstrated that GSDME deficiency facilitated breast cancer cell proliferation and colony formation while CDK7 inhibition impaired the ability of breast cancer proliferation and colony formation. We proposed a novel relationship in which CDK7 inhibition enhances the expression of GSDME in a p53-dependent pathway. We further verified that p53 and GSDME expressions are positively correlated and that p53 downregulation obstructs GSDME protein expression enhancement and CDK7-induced breast cancer cell survival suppression. These findings may reveal an anti-cancer mechanism of CDK7 inhibition.

## Materials and Methods

### Cell Line and Cell Culture

Cell lines MCF-7, HS578T, MB231, ZR-75-1, MCF10A and 293T were purchased from the Cell Bank of the Type Culture Collection of the Chinese Academy of Sciences (Shanghai, China). The cells were cultured in DMEM (Hyclone, Logan, United States) supplemented with 10% fetal bovine serum (FBS, Biological Industries, United States). MB231 and ZR-75-1 cells were cultured in RPMI medium (Hyclone, Logan, United States) supplemented with 10 and 15% FBS, respectively. MCF10A cells were cultured in DMEM/F-12 medium (GIBCO, 31330038) supplemented with 5% Donor Horse Serum (DHS, Biological Industries, 04-004-1B), 20 ng/ml EGF (Sigma, E9644), 0.5 μg/ml hydrocortisone (Sigma, H0888), 100 ng/ml cholera toxin (Sigma, C8052), and 10 μg/ml insulin (Sigma, I1882). All media contained 1% penicillin/streptomycin (Invitrogen, Carlsbad, CA) and were cultured under a humidified atmosphere of 5% carbon dioxide at 37°C.

### Reagents and Antibodies

THZ1 (HY-80013), LDC4297 **(**HY-12653**)**, nutlin-3 (HY-50696), and pifithrin-β (PFT β) (HY-16702) were purchased from MedChemExpress (Monmouth Junction, NJ, United States). The primary antibodies were antibodies against GAPDH (Santa Cruz, CA, United States), DFNA5/GSDME (Abcam, Cambridge, United Kingdom), CDK7 (Cell Signaling Technology, Danvers, MA, United States), and p53 (Proteintech, China). The secondary antibodies were anti-rabbit IgG (7074) and anti-mouse IgG (7076) (Cell Signaling Technology, Beverly, MA). All primary and secondary antibodies were diluted by TBS.

### Cell Viability Assay

MCF-7 and ZR-75-1 cells were seeded at a density of 6,000 cells/well, with 100 μl medium, in 96-well plates. After a 24 h incubation to allow adherence, the original medium was replaced with 100 μl same medium containing different treatment concentrations, and the cells were incubated further. We tested for cell viability at 24 and 48 h. The cells were incubated with 100 μl cell culture medium and 10 µl CCK8 (Abcam, Cambridge, United Kingdom) at 37°C for 2 h before assessing the OD. The absorbance was read at a wavelength of 450 nM using a BioTek ELISA reader (Winooski, VT, United States). All tests were independently repeated three times.

### Lentivirus Preparation

The plasmids pRSV-Rev (12253), pMDLg/pRRE (12251), and pCMV-VSV-G (8454) were purchased from Addgene (Cambridge, MA, United States). The lentiviral vector pLKO.1 was obtained from Generay Biotech (Shanghai, China). shRNA targeting CDK7 (sequence: 5′-CCG​GGC​TGT​AGA​AGT​GAG​TTT​GTA​ACT​CGA​GTT​ACA​AAC​TCA​CTT​CTA​CAG​CTT​TTT-3′) was purchased from Sigma-Aldrich (United States). The packaged viral vectors were added into the culture medium of 293T cells, and after 48 h, the supernatant of the 293T cells was collected and purified through 0.45 µm membranes. Lentiviral transfection was performed as previously reported (Peng et al., 2021), to downregulate the expression of related proteins in breast cancer cells. The effect of shRNA was assessed using western blotting.

### Transfection Assay

MCF-7 cells were seeded with 2 ml medium in six-well plates, and after reaching 70% confluence, they were transfected with GSDME shRNA plasmids (GenePharma, Shanghai, China), using Lipofectamine 3000 (Thermo Fisher Scientific, United States). A lipo3000 diluent was prepared by diluting 5 μl/well lipo3000 with 125 μl/well Opti-MEM medium and a master mix of DNA by diluting 2 μg/well DNA plasmids with a mixture of 125μl/well Opti-MEM medium + 5 μl/well lipo3000. The latter was added to the former, and the compound was incubated for 10 min at room temperature before being applied to the cells, which were then incubated for another 48 h.

MCF-7 cells were seeded with 2 ml medium in six-well plates, and after reaching 70% confluence, they were transfected with negative control or p53 siRNAs (GenePharma, Shanghai, China), using Lipofectamine 3000. A lipo3000 diluent was prepared by diluting 5 μl/well lipo3000 with 125 μl/well Opti-MEM medium and a master mix of siRNA by diluting 3 μg/well siRNA plasmids with 125 μl/well Opti-MEM medium. The latter was added to the former, and the compound was incubated for 10 min at room temperature before being applied to the cells, which were then incubated for another 48 h.

Transfections were conducted according to the Lipofectamine 3000 manufacturer’s instructions. The effect of transfection was assessed using western blotting.

### Colony Formation Assay

MCF-7 and ZR-75-1 cells were seeded at approximately 6,000 cells/well and 10,000 cells/well, respectively, with 2 ml medium, in six-well plates. 24 h later, the original medium was replaced with 2 ml same medium containing different treatment concentrations, and the cells were incubated for 7 days. For the colony formation assay of MCF-7 cells with GSDME or CDK7 downregulation treatment, relevant genes were downregulated prior to seeding. Then, 6,000 cells/well of MCF-7 cells were incubated in 2 ml medium for 7 days. All cells which would be utilized to conduct the colony formation assay were washed with cold phosphate-buffered saline (PBS), fixed with 4% paraformaldehyde for 20 min, and stained with 0.1% crystal violet solution for 15 min at room temperature. The plates were then washed with double-distilled water and air-dried. The number of colonies on each plate was counted to assess the cell colony formation ability. All tests were independently repeated three times.

### Apoptosis Assay

MCF-7 cells were seeded at approximately 120,000 cells/well with 2 ml medium in six-well plates. After a 24 h incubation, the original medium was replaced with 2 ml of the same medium containing different treatment concentrations. Following a 24 h incubation, the cells were washed twice with PBS, trypsinized softly, and pelleted by centrifugation at 500 g for 5 min at 4°C. The cells were resuspended in cold PBS, collected by centrifugation at 500 g, and resuspended in 100 μl of cold binding buffer. Then, each group was treated with 5 μl annexin V-FITC+5 μl PI. The cells were incubated with fluorochrome for 15 min at room temperature. 400 μl of binding buffer was added to each group of cells, before apoptosis was detected using flow cytometry. The Annexin V-FITC/PI Cell Apoptosis Detection Kit was purchased from TransGen (Beijing, China); the flow cytometry machine was obtained from Thermo Fisher Scientific (United States).

### Western Blotting

MCF-7 and ZR-75-1 cells were seeded at 12,000 cells/well with 2 ml medium in six-well plates and allowed to adhere for 24 h. The cells were then treated with different treatment concentrations or transfected, washed twice with PBS, and lysed with RIPA lysis buffer for 10 min on ice. The collected cell lysate was centrifuged at 14,000 *g* for 15 min at 4°C. Protein levels were quantified using a Pierce BCA kit (Thermo Fisher Scientific, United States). The supernatant was extracted before mixing with loading buffer, and the mixture was heated at 100°C for 10 min. Samples were added at 20 μg/well. Proteins of different sizes were separated on 10% polyacrylamide gels and transferred onto PVDF membranes. The membranes were sliced into pieces, blocked with 5% skim milk for 1 h at room temperature, and incubated with primary antibodies overnight at 4°C. After three TBST washing cycles, the PVDF membranes were incubated with secondary antibodies at room temperature for 2 h. Protein expression levels were detected using Pierce ECL reagent (Thermo Fisher Scientific, United States). All blots in this study were corrected for GAPDH expression levels, and all tests were independently repeated three times.

### Statistical Analysis

The differences between test groups were analyzed using GraphPad Prism software (version 8.0; GraphPad Software, La Jolla, CA, United States) and SPSS 22.0. Student’s t-test was used for statistical analyses, and statistical significance was set at *p* < 0.05. All experiments were repeated at least three times.

## Results

### CDK7 Inhibitors Affected MCF-7 Cell Survival

**FIGURE 1:**
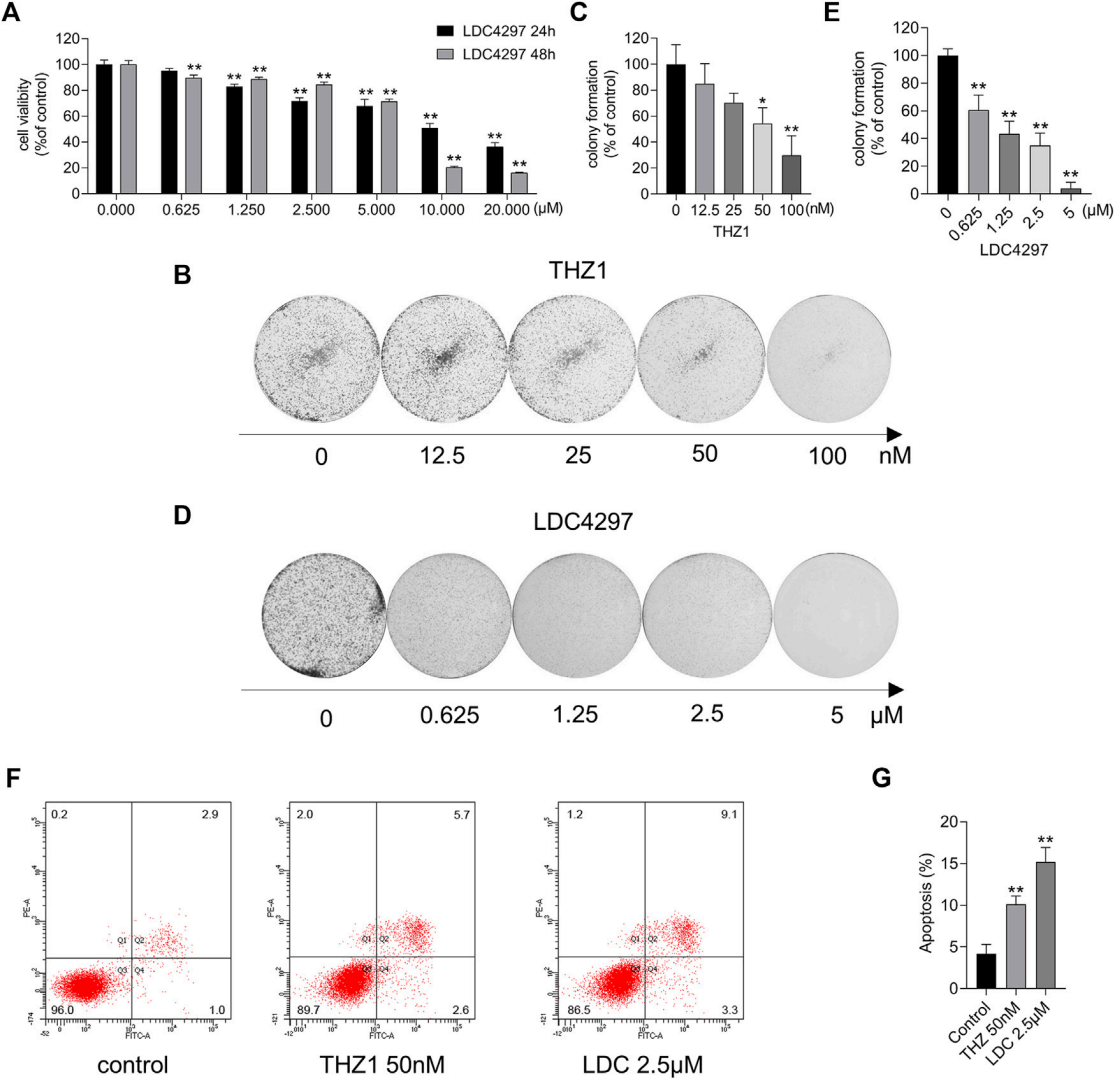
CDK7 inhibitors affected MCF-7 cell survival. **(A)** The cell viability was detected by the CCK8 assay after MCF-7 cells were treated with increasing concentrations of LDC4297 for 24 or 48 h separately. **(B, D)** The colony formation ability was detected after MCF-7 cells were treated with THZ1 or LDC4297. **(C, E)** Quantitation of colony formation ability in **(B)** and **(D)** separately. **(F)** Apoptosis was analyzed by flow cytometry after MCF-7 cells were treated with 50 nM THZ1 or LDC4297 2.5 μM for 24 h. **(G)** Quantity of apoptosis in **(F)**. Statistically significant: *p < 0.05; **p < 0.01.

To explore the effect of CDK7 inhibitors on breast cancer cells, we measured the viability of MCF-7 cells after incubation with increasing concentrations of THZ1 and LDC4297 for 24 and 48 h. The CCK8 results showed that although the survival of MCF-7 cells was not significantly affected by treatment with low concentrations of THZ1, the repressive effect could still be detected in a dose- and time-dependent manner, confirming previous results (Peng et al., 2021). As shown in Figure 1A, LDC4297 also suppressed tumor growth. The colony formation assay revealed that CDK7 inhibitors attenuated the tumor stem cell characteristics of MCF-7 cells (Figures 1B–E). Additionally, the results of the flow cytometry analysis confirmed that apoptosis occurred in MCF-7 cells treated for 24 h with THZ1 and LDC4297 (Figures 1F,G). Both THZ1 and LDC4297 suppressed MCF-7 cells by slowing down cell proliferation and inducing apoptosis, which implied the potential breast tumor suppressive effect of CDK7 inhibitors.

### Inhibiting CDK7 Upregulated the Expression of p53 and GSDME

**FIGURE 2:**
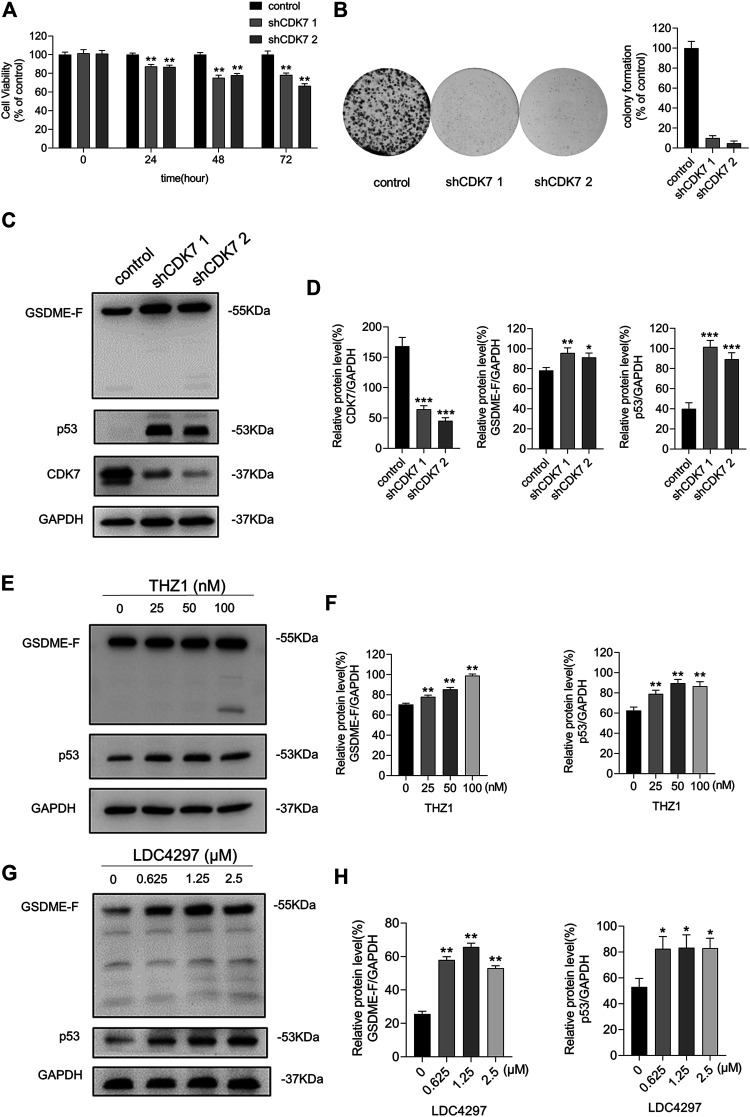
Inhibiting CDK7 upregulated the expression of p53 and GSDME. **(A)** The CCK8 assay was utilized to measure the viability of MCF-7 cells with CDK7 shRNA at 24, 48, and 72 h. **(B)** The colony formation assay was meant to test the ability of MCF-7 cells with CDK7 shRNA and control shRNA to form colony spots. **(C)** The silencing effect of CDK7 shRNA carried by lentiviruses was determined by western blot. **(D)** Quantity of CDK7, GSDME, and p53 in **(C)**. **(E)** Western blot was conducted to detect the varieties of GSDME and p53 protein levels in MCF-7 cells which were treated with THZ1 for 36 h. **(F)** Quantity of GSDME and p53 in **(E)**. **(G)** MCF-7 cells were treated with LDC4297 for 36 h. Western blot was meant for the varieties of GSDME and p53 protein levels in MCF-7 cell detection. **(H)** Quantity of GSDME and p53 in **(G)**. Statistically significant: *p < 0.05; **p < 0.01.

We transfected breast cancer cells with CDK7 shRNA lentiviruses to verify the anti-tumor effect of CDK7 inhibition. As shown in Figures 2A,B, the group with CDK7 shRNA vectors displayed decreased cell proliferation and fewer colony spots than the control group. In our exploration of the regulatory mechanism, expression levels of p53, a downstream effector of CDK7 (Figures 2C,D), indicated that CDK7 inhibition increased the p53 protein levels, confirming previous results (Peng et al., 2021).

Because Arakawa’s work demonstrated that GSDME might be a p53 target, we proceeded to detect GSDME expression levels during CDK7 inhibition. Interestingly, the protein level of GSDME was enhanced after CDK7 silencing (Figures 2C,D). This result prompted us to explore the internal control between CDK7 and GSDME and the possibility that GSDME contributed to the tumor suppressive effects of CDK7 inhibition. Treatment of MCF-7 cells with increasing concentrations of THZ1 or LDC4297 exhibited a gradual increase in p53 and GSDME expressions (Figures 2E–H). These results support a possible modulation relationship among CDK7, p53, and GSDME.

### Loss of GSDME Expression Increased Breast Cancer Cell Proliferation and Colony-Forming Capacity

**FIGURE 3:**
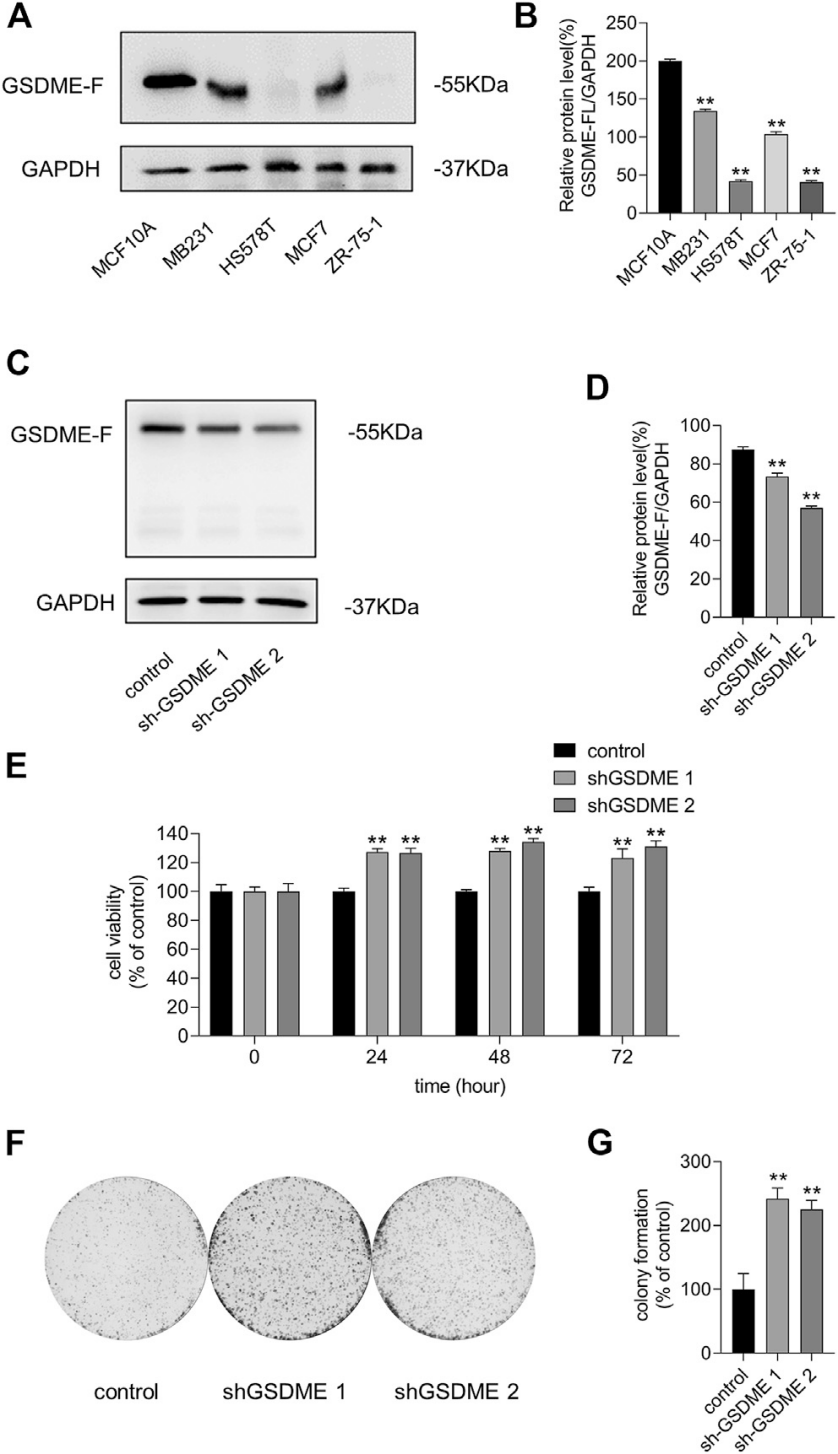
Loss of GSDME expression increased breast cancer cell proliferation and colony ability. **(A)** The protein level of GSDME was detected by western blot. **(B)** Quantity of GSDME in **(A)**. **(C)** Western blot was utilized to verify the silencing effects of GSDME shRNA plasmids in MCF-7 cells. **(D)** Quantity of GSDME in **(C)**. **(E)** Different time-point cell viability of MCF-7 cells after transfecting GSDME shRNA plasmids and shRNA of control was measured by the CCK8 assay. **(F)** Colony formation ability measurement of MCF-7 cells after transfecting shRNA of GSDME and shRNA of control. **(G)** Quantitation of colony formation ability in **(F)**. Statistically significant: *p < 0.05; **p < 0.01.

We performed western blotting to detect the GSDME protein levels in multiple breast cancer cell lines. We discovered that the protein level of GSDME was lower in breast cancer cells MB231, HS578T, MCF-7, and ZR-75-1 than in non-tumorigenic human mammary epithelial MCF10A cells (Figures 3A,B). This suggests that the neoplastic characteristics of breast cancer cells might be due in part to the loss of GSDME expression. We verified the impact of GSDME on MCF-7 cells by knocking down its expression (Figures 3C,D) and discovered GSDME downregulation promoted MCF-7 cell growth and colony formation (Figures 3E–G). These outcomes suggest that low expression of GSDME is required for both cell proliferation and colony formation of breast cancer cells. Thus, we hypothesized that GSDME expression elevation is a possible route for CDK7 inhibition to suppress breast cancer cell survival.

### GSDME Expression Was Mediated by p53 Activity

**FIGURE 4:**
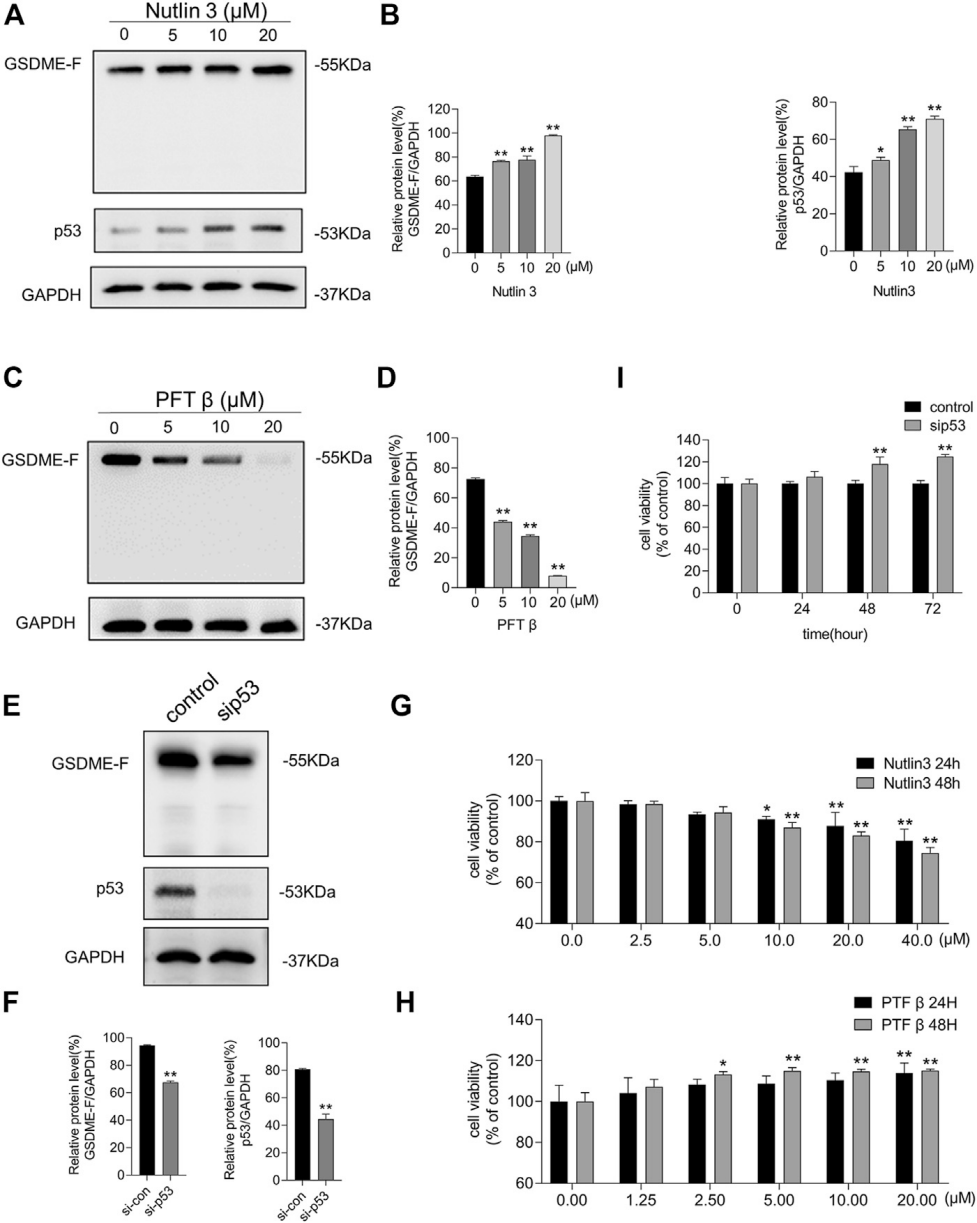
The expression of GSDME was mediated by p53 activity. **(A)** Western blot was conducted to display the expression of GSDME, p53, and GAPDH in MCF-7 cells treated with nutlin-3 for 48 h. **(B)** Quantity of GSDME and p53 in **(A)**. **(C)** Western blot was conducted to display the expression of GSDME and GAPDH in MCF-7 cells treated with PFT *β* for 48 h. **(D)** Quantity of GSDME in **(C)**. **(E)** Western blot was utilized to confirm the knockdown effect of p53 siRNA in MCF-7 cells. **(F)** Quantity of GSDME and p53 in MCF-7 cells after p53 siRNA transfection. **(G)** The CCK8 assay was used to measure the cell viability of MCF-7 cells treated with nutlin-3 for 24 and 48 h. **(H)** The CCK8 assay was used to measure the cell viability of MCF-7 cells treated with PFT *β* for 24 and 48 h. **(I)** MCF-7 cell viability with p53 siRNA transfection was detected by the CCK8 assay. Statistically significant: *p < 0.05; **p < 0.01.

Nutlin-3, an MDM2 inhibitor that leads to p53 accumulation and activation, and PFT β, a p53 interfering agent that reversibly blocks p53-dependent transcriptional activation but barely affects p53 mRNA levels, were used to verify whether p53 is required for the expression of GSDME. We treated MCF-7 cells with nutlin-3 for 48 h, which resulted in elevated p53 and GSDME protein levels (Figures 4A,B). We subsequently treated MCF-7 cells with PFT β, which resulted in decreased GSDME protein levels (Figures 4C,D). To investigate whether p53 underexpression would affect GSDME levels, we transfected siRNA to inhibit p53 expression. As shown in Figures 4E,F, siRNA-blocked p53 expression resulted in decreased GSDME protein levels. Thus, p53 accumulation led to upregulated GSDME protein expression, while p53 repression, *via* either siRNA interference or activity inhibitor, led to downregulated GSDME protein expression. Moreover, treatment with nutlin-3 reduced MCF-7 cell proliferation (Figure 4G), while treatment with PFT β or siRNA-induced p53 inhibition promoted MCF-7 cell proliferation (Figures 4H,I). These results suggest that GSDME participates in the modulation of p53, affecting breast cancer cell survival.

### p53 Inhibitor Blocked the Anti-Tumor Effects of CDK7 Inhibitors

**FIGURE 5:**
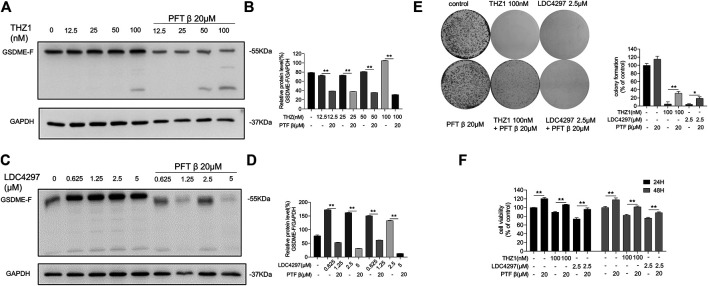
p53 inhibitor blocked the anti-cancer effects of CDK7 inhibitors. **(A)** MCF-7 cells were firstly given PFT *β* for 6 h and then treated with THZ1 for 36 h. Western blot was used to display the GSDME protein level. **(B)** Quantity of GSDME in **(A)**. **(C)** MCF-7 cells were firstly given PFT *β* for 6 h and then treated with LDC4297 for 36 h. Western blot was used to display the GSDME protein level. **(D)** Quantity of GSDME in **(C)**. **(E)** The colony formation assay was performed to detect the colony formation ability of MCF-7 cells given PFT *β* for 6 h before treated with THZ1 or LDC4297. **(F)** MCF-7 cells were firstly given PFT *β* for 6 h and then treated with THZ1 or LDC for 24 or 48 h separately. The CCK8 assay was used to measure the MCF-7 cell viability. Statistically significant: *p < 0.05; **p < 0.01.

To explore the possible effect of p53 activity on GSDME expression, we determined whether p53 bridged the modulation of GSDME by CDK7. We first pre-treated MCF-7 cells with PFT β for 6 h to obstruct the *p53* pathway. We then applied increasing concentrations of THZ1 or LDC4297 to both PFTβ pre–treated and untreated MCF-7 cells. GSDME production in cells pre-treated with PFT β remained low, as opposed to their untreated counterparts, in which GSDME production increased (Figures 5A–D). These results indicated that when p53 activity was repressed, the elevation of GSDME protein levels induced by CDK7 inhibition was reversed. Therefore, CDK7 likely regulates GSDME via a p53-dependent pathway.

In addition, MCF-7 cell proliferation and colony formation capacity inhibited by CDK7 were partially de-repressed by PFT *β* pre-addition (Figures 5E,F). As the results indicate, without functional p53 participation, the breast cancer cell suppression effect and GSDME protein level elevation induced by CDK7 inhibitors were limited.

### Inhibiting CDK7 Enhanced GSDME Expression in ZR-75-1 Cells

**FIGURE 6:**
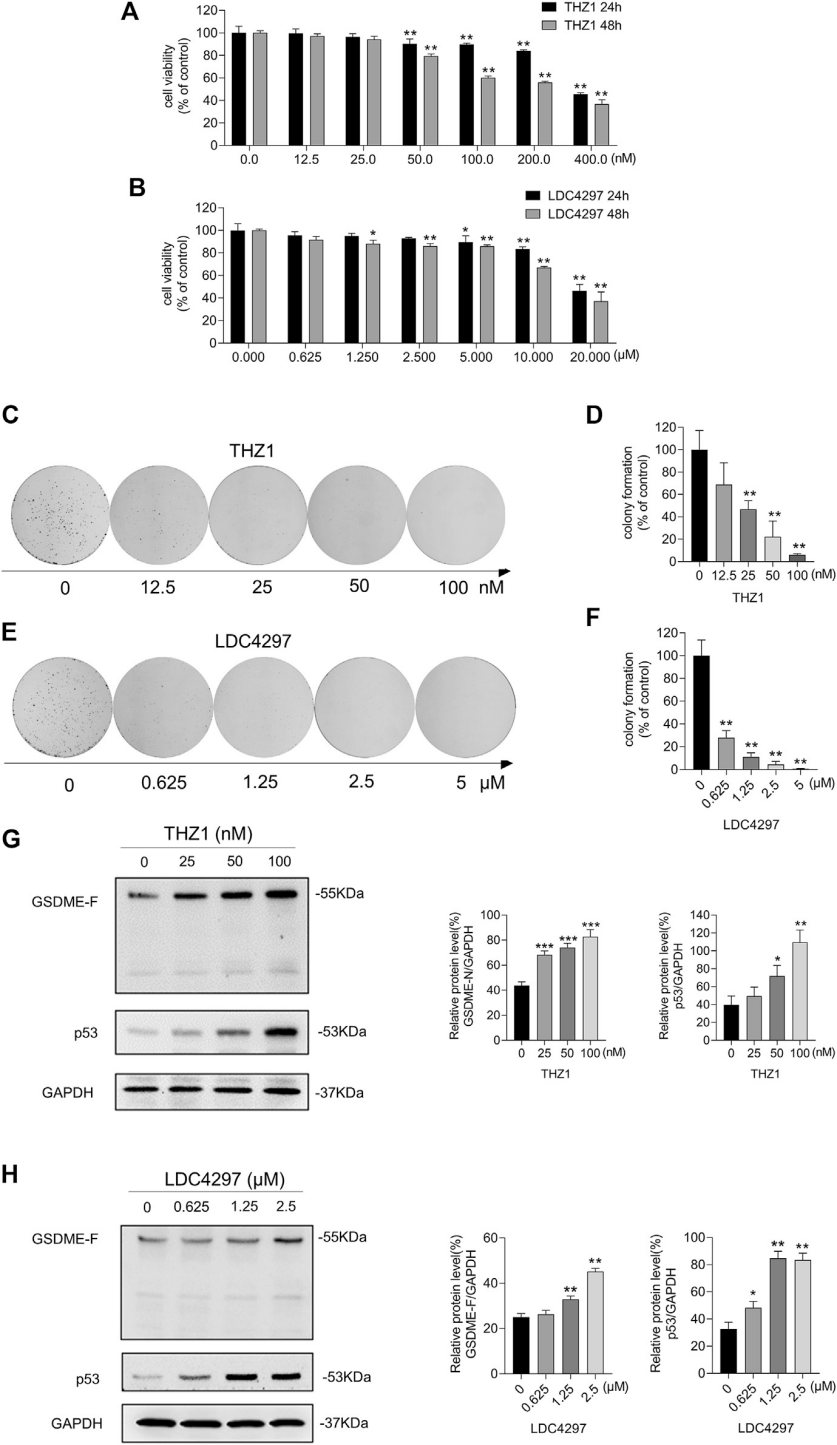
Inhibiting CDK7 enhanced GSDME expression in ZR-75-1 cells. **(A, B)** The CCK8 assay was meant for detecting the viability of ZR-75-1 cells after being treated with increasing concentrations of THZ1 or LDC4297 for 24 and 48 h. **(C, E)** The colony formation ability was detected after ZR-75-1 cells were treated with THZ1 or LDC4297. **(D, F)** Quantitation of colony formation ability in **(C)** and **(E)** separately. **(G)** Western blot was meant to detect GSDME, p53, and GAPDH protein levels in ZR-75-1 cells which were treated with THZ1 for 48 h. **(H)** Western blot was meant to detect GSDME, p53, and GAPDH protein levels in ZR-75-1 cells which were treated with LDC4297 for 48 h. Statistically significant: *p < 0.05; **p < 0.01.

The ZR-75-1 cell line is a type of breast cancer cell with lower GSDME expression than MCF-7 cells and lacking caspase-3 expression. We treated ZR-75-1 cells with THZ1 and LDC4297 and observed the impairment of cell viability (Figures 6A,B) and measured GSDME and p53 protein levels. ZR-75-1 cells treated with the CDK inhibitors produced fewer and smaller colony spots (Figures 6C–F) and, similarly to MCF-7 cells, exhibited increased GSDME and p53 protein levels (Figures 6G,H). These results suggest that CDK7 inhibition suppresses breast cancer cells via elevation of p53 and GSDME protein levels.

## Discussion

Loss of CDK activity regulation is a common characteristic of many tumors, and CDK inhibition provides a potent approach to tumor suppression. At present, CDK4/6 inhibitors have been designed to target the G1-to-S phase transition, and some have been applied in clinical settings (O'Leary et al., 2016). Upstream of CDK4/6, CDK7 plays a crucial role in regulating the cell cycle *via* activation of CDKs and RNAP II–mediated transcription (Ganuza et al., 2012; Schachter et al., 2013). Notably, its expression is implicated in poor prognosis. Here, we explored the CDK7 inhibition activity of THZ1 and LDC4297 on MCF-7 and ZR-75-1 breast cancer cells. Because caspase-3 is a GSDME cleavage trigger, and this study aimed to detect GSDME in full-length form, ZR-71-1 was chosen for its lack of both caspase 3 expression and *p53* mutations (Keyomarsi and Pardee, 1993; Devarajan et al., 2002). The repression of breast cancer cell proliferation and colony formation was observed following CDK7 inhibition treatment (Figures 1, 6), identifying CDK7 inhibitors as potential agents for breast cancer treatment.

Studies confirm CDK7 silencing blocks RNAP II-CTD phosphorylation (Galbraith et al., 2019), and RNAP II-hypophosphorylation–induced suppression of transcription initiation and elongation can elevate p53 protein levels and accumulation in the nucleus by phosphorylating the p53 Ser-15 site (Derheimer et al., 2007). As has been shown in RKO and LNCaP cells (Ko et al., 1997), CDK7 controls p53 activation and accumulation. Studies consistently demonstrate that CDK7 inhibitors, such as THZ1, BS-181, and YKL-5-124, block RNAP II phosphorylation and induce p53 activation and intracellular p53 protein accumulation (Nilson et al., 2015; Wang et al., 2016b; Zhang et al., 2017; Zhong et al., 2018; Olson et al., 2019). Here, we demonstrate that both CDK7 transcriptional level obstruction and CDK7 inhibitors, THZ1 and LDC4297, increase p53 protein levels in MCF-7 and ZR-75-1 cells (Figures 2, 6). These outcomes may be due to CDK7’s role as a component of TFIIH, inhibition of which obstructs the RNAP II phosphorylation process. Additionally, RNAP II blockage can trigger the p53 response to harmful stimuli and facilitate p53 accumulation (Ljungman et al., 1999; Wang et al., 2015). Because THZ1 inhibits CDK7 via covalent binding rather than downregulation of CDK7 expression, continued CDK7 expression does not suppress p53 accumulation. Because p53 is a well-known tumor suppressor present in a wide range of cancer cells and modulates the transcription of various downstream genes, we hypothesized that p53 protein level elevation in breast cancer cells possibly presents a viable method of breast cancer treatment *via* CDK7 inhibition.

Certain p53 mutations lose the regulatory effect of GSDME expression (Masuda et al., 2006). Our study demonstrates that blocking p53 activity also blocks CDK7-inhibition–induced GSDME overexpression (Figures 5A,C) and partially restores breast cancer cell proliferation and colony formation capacity from the suppressive effect of CDK7 inhibition. These results suggest that the effect of CDK7 inhibitors on breast cancer cells is partially dependent on p53 status, which should be considered an effective indicator before CDK7 inhibition therapy application and a prognostic indicator after therapy completion.

Knockout of DFNA5 facilitates the resistance of melanoma cells to etoposide (Lage et al., 2001). Here, we consistently demonstrated that GSDME downregulation facilitated breast cancer cell growth and colony formation (Figures 3E–G). Furthermore, a potential p53-binding sequence is located in intron 1 of DFNA5, identifying it as a possible p53 target gene (Masuda et al., 2006) (Figures 4A,C,E). Hence, we explored the association between CDK7 and GSDME and found that GSDME expression was inversely related to CDK7 levels via p53 mediation (Figures 2, 5, 6). This finding suggests CDK7 inhibitors induce tumor suppressor GSDME expression to realize its anti-tumor effect. Transfection of DFNA5 into HepG2 cells leads to G2-to-M phase arrest and Fas and caspase-8 overexpression. Subsequently, p53 transcription activation produces THZ1 sensitivity in the tumor cells, triggering an extrinsic apoptotic pathway and DR5 expression, which, in turn, induces caspase-8 cleavage (Wang et al., 2013; Kalan et al., 2017). A collective view with the results of our study indicates the p53–GSDME relationship plays a possible and important role in the cell death induction process. Because p53 accumulation leads to GSDME upregulation (Figure 4A) which induces caspase-8 expression, extrinsic-pathway activation and caspase-8 cleavage are possibly amplified by relative protein level elevation. Additionally, the product of GSDME cleavage, the GSDME N-terminal domain, exhibits pore-forming activity and an affinity to plasma membranes, including the cell and mitochondrial membranes. The N-terminal domain has been reported to permeabilize the mitochondria to release cyt c and facilitate mitochondrial ROS accumulation, which contribute to cell death induction. Hence, GSDME bridged the extrinsic and intrinsic apoptotic pathways by destroying mitochondrial integrity, leading to cyt c leakage, followed by caspase-3 activation amplification (Rogers et al., 2019). Here, we suggest that GSDME acts as a tumor suppressor because of its multiple functions in cell death modulation by inducing pyroptosis, potentiating caspase-3 cleavage with mitochondrial apoptotic pathway activation, and elevating extrinsic death protein expression.

In this study, we investigated whether CDK7 inhibition was an effective anti-tumor option and whether GSDME expression was associated with CDK7 levels. We also demonstrated that the GSDME deletion contributed to breast cancer cell proliferation and colony formation. After validation in two types of breast cancer cells, we hypothesized that CDK7 inhibition may elevate GSDME protein levels under p53 modulation. However, not all types of breast cancer were utilized to confirm this conjecture; thus, much work remains. To our knowledge, our report is the first to describe the relationship among CDK7, p53, and GSDME in breast cancer cells and, thus, facilitates a deeper understanding of CDK7 activity and provides a novel anti-tumor target.

## Data Availability

The original contributions presented in the study are included in the article/supplementary material, and further inquiries can be directed to the corresponding author.

## References

[B1] Akhtar M. S. Heidemann M. Tietjen J. R. Zhang D. W. Chapman R. D. Eick D. (2009). TFIIH Kinase Places Bivalent marks on the Carboxy-Terminal Domain of RNA Polymerase II. Mol. Cel. 34 (3), 387–393. 10.1016/j.molcel.2009.04.016 PMC2757088 19450536

[B2] Akino K. Toyota M. Suzuki H. Imai T. Maruyama R. Kusano M. (2007). Identification of DFNA5 as a Target of Epigenetic Inactivation in Gastric Cancer. Cancer Sci. 98 (1), 88–95. 10.1111/j.1349-7006.2006.00351.x 17083569 PMC11158324

[B3] Ali S. Heathcote D. A. Kroll S. H. B. Jogalekar A. S. Scheiper B. Patel H. (2009). The Development of a Selective Cyclin-dependent Kinase Inhibitor that Shows Antitumor Activity. Cancer Res. 69 (15), 6208–6215. 10.1158/0008-5472.can-09-0301 19638587 PMC2875168

[B4] Attia Y. M. Shouman S. A. Salama S. A. Ivan C. Elsayed A. M. Amero P. (2020). Blockade of CDK7 Reverses Endocrine Therapy Resistance in Breast Cancer. Int. J. Mol. Sci. 21 (8). 10.3390/ijms21082974 PMC7215326 32340192

[B5] Christensen C. L. Kwiatkowski N. Abraham B. J. Carretero J. Al-Shahrour F. Zhang T. (2014). Targeting Transcriptional Addictions in Small Cell Lung Cancer with a Covalent CDK7 Inhibitor. Cancer cell 26 (6), 909–922. 10.1016/j.ccell.2014.10.019 25490451 PMC4261156

[B6] Croes L. Beyens M. Fransen E. Ibrahim J. Vanden Berghe W. Suls A. (2018). Large-scale Analysis of DFNA5 Methylation Reveals its Potential as Biomarker for Breast Cancer. Clin. epigenetics 10, 51. 10.1186/s13148-018-0479-y 29682089 PMC5896072

[B7] Deng J. L. Xu Y. H. Wang G. (2019). Identification of Potential Crucial Genes and Key Pathways in Breast Cancer Using Bioinformatic Analysis. Front. Genet. 10, 695. 10.3389/fgene.2019.00695 31428132 PMC6688090

[B8] Derheimer F. A. O'Hagan H. M. Krueger H. M. Hanasoge S. Paulsen M. T. Ljungman M. (2007). RPA and ATR Link Transcriptional Stress to P53. Proc. Natl. Acad. Sci. 104 (31), 12778–12783. 10.1073/pnas.0705317104 17616578 PMC1937543

[B9] Devarajan E. Sahin A. A. Chen J. S. Krishnamurthy R. R. Aggarwal N. Brun A.-M. (2002). Down-regulation of Caspase 3 in Breast Cancer: a Possible Mechanism for Chemoresistance. Oncogene 21 (57), 8843–8851. 10.1038/sj.onc.1206044 12483536

[B10] Diab S. Yu M. Wang S. (2020). CDK7 Inhibitors in Cancer Therapy: The Sweet Smell of Success? J. Med. Chem. 63 (14), 7458–7474. 10.1021/acs.jmedchem.9b01985 32150405

[B11] Fisher R. P. (2012). The CDK Network: Linking Cycles of Cell Division and Gene Expression. Genes. cancer 3 (11-12), 731–738. 10.1177/1947601912473308 23634260 PMC3636752

[B12] Galbraith M. D. Bender H. Espinosa J. M. (2019). Therapeutic Targeting of Transcriptional Cyclin-dependent Kinases. Transcription 10 (2), 118–136. 10.1080/21541264.2018.1539615 30409083 PMC6602565

[B13] Ganuza M. Sáiz-Ladera C. Cañamero M. Gómez G. Schneider R. Blasco M. A. (2012). Genetic Inactivation of Cdk7 Leads to Cell Cycle Arrest and Induces Premature Aging Due to Adult Stem Cell Exhaustion. EMBO J. 31 (11), 2498–2510. 10.1038/emboj.2012.94 22505032 PMC3365431

[B14] Hutterer C. Eickhoff J. Milbradt J. Korn K. Zeitträger I. Bahsi H. (2015). A Novel CDK7 Inhibitor of the Pyrazolotriazine Class Exerts Broad-Spectrum Antiviral Activity at Nanomolar Concentrations. Antimicrob. Agents Chemother. 59 (4), 2062–2071. 10.1128/aac.04534-14 25624324 PMC4356785

[B15] Jiang L. Huang R. Wu Y. Diao P. Zhang W. Li J. (2019). Overexpression of CDK7 Is Associated with Unfavourable Prognosis in Oral Squamous Cell Carcinoma. Pathology 51 (1), 74–80. 10.1016/j.pathol.2018.10.004 30473182

[B16] Kalan S. Amat R. Schachter M. M. Kwiatkowski N. Abraham B. J. Liang Y. (2017). Activation of the P53 Transcriptional Program Sensitizes Cancer Cells to Cdk7 Inhibitors. Cel Rep. 21 (2), 467–481. 10.1016/j.celrep.2017.09.056 PMC5687273 29020632

[B17] Keyomarsi K. Pardee A. B. (1993). Redundant Cyclin Overexpression and Gene Amplification in Breast Cancer Cells. Proc. Natl. Acad. Sci. 90 (3), 1112–1116. 10.1073/pnas.90.3.1112 8430082 PMC45821

[B18] Kim M. S. Chang X. Yamashita K. Nagpal J. K. Baek J. H. Wu G. (2008). Aberrant Promoter Methylation and Tumor Suppressive Activity of the DFNA5 Gene in Colorectal Carcinoma. Oncogene 27 (25), 3624–3634. 10.1038/sj.onc.1211021 18223688

[B19] Kim M. S. Lebron C. Nagpal J. K. Chae Y. K. Chang X. Huang Y. (2008). Methylation of the DFNA5 Increases Risk of Lymph Node Metastasis in Human Breast Cancer. Biochem. biophysical Res. Commun. 370 (1), 38–43. 10.1016/j.bbrc.2008.03.026 PMC3094717 18346456

[B20] Ko L. J. Shieh S. Y. Chen X. Jayaraman L. Tamai K. Taya Y. (1997). p53 Is Phosphorylated by CDK7-Cyclin H in a p36MAT1-dependent Manner. Mol. Cel. Biol. 17 (12), 7220–7229. 10.1128/mcb.17.12.7220 PMC232579 9372954

[B21] Kwiatkowski N. Zhang T. Rahl P. B. Abraham B. J. Reddy J. Ficarro S. B. (2014). Targeting Transcription Regulation in Cancer with a Covalent CDK7 Inhibitor. Nature 511 (7511), 616–620. 10.1038/nature13393 25043025 PMC4244910

[B22] Laer L. V. Huizing E. H. Verstreken M. Zuijlen D. v. Wauters J. G. Bossuyt P. J. (1998). Nonsyndromic Hearing Impairment Is Associated with a Mutation in DFNA5. Nat. Genet. 20 (2), 194–197. 10.1038/2503 9771715

[B23] Lage H. Helmbach H. Grottke C. Dietel M. Schadendorf D. (2001). DFNA5 (ICERE-1) Contributes to Acquired Etoposide Resistance in Melanoma Cells. FEBS Lett. 494 (1-2), 54–59. 10.1016/s0014-5793(01)02304-3 11297734

[B24] Larochelle S. Amat R. Glover-Cutter K. Sansó M. Zhang C. Allen J. J. (2012). Cyclin-dependent Kinase Control of the Initiation-To-Elongation Switch of RNA Polymerase II. Nat. Struct. Mol. Biol. 19 (11), 1108–1115. 10.1038/nsmb.2399 23064645 PMC3746743

[B25] Li B. Ni Chonghaile T. Fan Y. Madden S. F. Klinger R. O'Connor A. E. (2017). Therapeutic Rationale to Target Highly Expressed CDK7 Conferring Poor Outcomes in Triple-Negative Breast Cancer. Cancer Res. 77 (14), 3834–3845. 10.1158/0008-5472.can-16-2546 28455421

[B26] Ljungman M. Zhang F. Chen F. Rainbow A. J. McKay B. C. (1999). Inhibition of RNA Polymerase II as a Trigger for the P53 Response. Oncogene 18 (3), 583–592. 10.1038/sj.onc.1202356 9989808

[B27] Lu P. Geng J. Zhang L. Wang Y. Niu N. Fang Y. (2019). THZ1 Reveals CDK7-dependent Transcriptional Addictions in Pancreatic Cancer. Oncogene 38 (20), 3932–3945. 10.1038/s41388-019-0701-1 30692639

[B28] Masuda Y. Futamura M. Kamino H. Nakamura Y. Kitamura N. Ohnishi S. (2006). The Potential Role of DFNA5, a Hearing Impairment Gene, in P53-Mediated Cellular Response to DNA Damage. J. Hum. Genet. 51 (8), 652–664. 10.1007/s10038-006-0004-6 16897187

[B29] Mayer E. L. (2015). Targeting Breast Cancer with CDK Inhibitors. Curr. Oncol. Rep. 17 (5), 443. 10.1007/s11912-015-0443-3 25716100

[B30] Nilson K. A. Guo J. Turek M. E. Brogie J. E. Delaney E. Luse D. S. (2015). THZ1 Reveals Roles for Cdk7 in Co-transcriptional Capping and Pausing. Mol. Cel. 59 (4), 576–587. 10.1016/j.molcel.2015.06.032 PMC4546572 26257281

[B31] O'Leary B. Finn R. S. Turner N. C. (2016). Treating Cancer with Selective CDK4/6 Inhibitors. Nat. Rev. Clin. Oncol. 13 (7), 417–430. 10.1038/nrclinonc.2016.26 27030077

[B32] Olson C. M. Liang Y. Leggett A. Park W. D. Li L. Mills C. E. (2019). Development of a Selective CDK7 Covalent Inhibitor Reveals Predominant Cell-Cycle Phenotype. Cel Chem. Biol. 26 (6), 792–803. 10.1016/j.chembiol.2019.02.012 PMC6588464 30905681

[B33] Patel H. Abduljabbar R. Lai C.-F. Periyasamy M. Harrod A. Gemma C. (2016). Expression of CDK7, Cyclin H, and MAT1 Is Elevated in Breast Cancer and Is Prognostic in Estrogen Receptor-Positive Breast Cancer. Clin. Cancer Res. 22 (23), 5929–5938. 10.1158/1078-0432.ccr-15-1104 27301701 PMC5293170

[B34] Peng J. Yang M. Bi R. Wang Y. Wang C. Wei X. (2021). Targeting Mutated P53 Dependency in Triple-Negative Breast Cancer Cells through CDK7 Inhibition. Front. Oncol. 11, 664848. 34109118 10.3389/fonc.2021.664848 PMC8183379

[B35] Rogers C. Erkes D. A. Nardone A. Aplin A. E. Fernandes-Alnemri T. Alnemri E. S. (2019). Gasdermin Pores Permeabilize Mitochondria to Augment Caspase-3 Activation during Apoptosis and Inflammasome Activation. Nat. Commun. 10 (1), 1689. 10.1038/s41467-019-09397-2 30976076 PMC6459836

[B36] Rogers C. Fernandes-Alnemri T. Mayes L. Alnemri D. Cingolani G. Alnemri E. S. (2017). Cleavage of DFNA5 by Caspase-3 during Apoptosis Mediates Progression to Secondary Necrotic/pyroptotic Cell Death. Nat. Commun. 8, 14128. 10.1038/ncomms14128 28045099 PMC5216131

[B37] Schachter M. M. Fisher R. P. (2013). The CDK-Activating Kinase Cdk7. Cell Cycle 12 (20), 3239–3240. 10.4161/cc.26355 24036541 PMC3885630

[B38] Schachter M. M. Merrick K. A. Larochelle S. Hirschi A. Zhang C. Shokat K. M. (2013). A Cdk7-Cdk4 T-Loop Phosphorylation cascade Promotes G1 Progression. Mol. Cel. 50 (2), 250–260. 10.1016/j.molcel.2013.04.003 PMC3677717 23622515

[B39] Sun B. Mason S. Wilson R. C. Hazard S. E. Wang Y. Fang R. (2020). Inhibition of the Transcriptional Kinase CDK7 Overcomes Therapeutic Resistance in HER2-Positive Breast Cancers. Oncogene 39 (1), 50–63. 10.1038/s41388-019-0953-9 31462705 PMC6937212

[B40] Sung H. Ferlay J. Siegel R. L. Laversanne M. Soerjomataram I. Jemal A. (2021). Global Cancer Statistics 2020: GLOBOCAN Estimates of Incidence and Mortality Worldwide for 36 Cancers in 185 Countries. CA A. Cancer J. Clin. 71 (3), 209–249. 10.3322/caac.21660 33538338

[B41] Thompson D. A. Weigel R. J. (1998). Characterization of a Gene that Is Inversely Correlated with Estrogen Receptor Expression (ICERE-1) in Breast Carcinomas. Eur. J. Biochem. 252 (1), 169–177. 10.1046/j.1432-1327.1998.2520169.x 9523727

[B42] VanArsdale T. Boshoff C. Arndt K. T. Abraham R. T. (2015). Molecular Pathways: Targeting the Cyclin D-Cdk4/6 Axis for Cancer Treatment. Clin. Cancer Res. 21 (13), 2905–2910. 10.1158/1078-0432.ccr-14-0816 25941111

[B43] Wang B.-Y. Liu Q.-Y. Cao J. Chen J.-W. Liu Z.-S. (2016). Selective CDK7 Inhibition with BS-181 Suppresses Cell Proliferation and Induces Cell Cycle Arrest and Apoptosis in Gastric Cancer. Drug. Des. Devel. Ther. 10, 1181–1189. 10.2147/dddt.s86317 PMC4801149 27042010

[B44] Wang C.-J. Tang L. Shen D.-W. Wang C. Yuan Q.-Y. Gao W. (2013). The Expression and Regulation of DFNA5 in Human Hepatocellular Carcinoma DFNA5 in Hepatocellular Carcinoma. Mol. Biol. Rep. 40 (12), 6525–6531. 10.1007/s11033-013-2581-8 24154762

[B45] Wang Q. Li M. Zhang X. Huang H. Huang J. Ke J. (2016). Upregulation of CDK7 in Gastric Cancer Cell Promotes Tumor Cell Proliferation and Predicts Poor Prognosis. Exp. Mol. Pathol. 100 (3), 514–521. 10.1016/j.yexmp.2016.05.001 27155449

[B46] Wang Y. Gao W. Shi X. Ding J. Liu W. He H. (2017). Chemotherapy Drugs Induce Pyroptosis through Caspase-3 Cleavage of a Gasdermin. Nature 547 (7661), 99–103. 10.1038/nature22393 28459430

[B47] Wang Y. Zhang T. Kwiatkowski N. Abraham B. J. Lee T. I. Xie S. (2015). CDK7-dependent Transcriptional Addiction in Triple-Negative Breast Cancer. Cell 163 (1), 174–186. 10.1016/j.cell.2015.08.063 26406377 PMC4583659

[B48] Zhang Z. Peng H. Wang X. Yin X. Ma P. Jing Y. (2017). Preclinical Efficacy and Molecular Mechanism of Targeting CDK7-dependent Transcriptional Addiction in Ovarian Cancer. Mol. Cancer Ther. 16 (9), 1739–1750. 10.1158/1535-7163.mct-17-0078 28572168

[B49] Zhang Z. Zhang Y. Xia S. Kong Q. Li S. Liu X. (2020). Gasdermin E Suppresses Tumour Growth by Activating Anti-tumour Immunity. Nature 579 (7799), 415–420. 10.1038/s41586-020-2071-9 32188940 PMC7123794

[B50] Zhong L. Yang S. Jia Y. Lei K. (2018). Inhibition of Cyclin‐dependent Kinase 7 Suppresses Human Hepatocellular Carcinoma by Inducing Apoptosis. J. Cel. Biochem. 119 (12), 9742–9751. 10.1002/jcb.27292 30145799

